# Brain precursor cells' failure in neurodegeneration

**DOI:** 10.18632/aging.101593

**Published:** 2018-10-20

**Authors:** Tamir Ben-Hur, Nina Fainstein

**Affiliations:** 1 Department of Neurology, Hadassah – Hebrew University Medical Center, Jerusalem, Israel

**Keywords:** neural stem cells, Alzheimer's disease

Extensive research has focused traditionally on the various pathogenic factors which fuel neurodegenerative processes, and lead to progressive loss of brain neurons, a principal pathological feature of dementing diseases. For example, vast research on Alzheimer's disease (AD) studied the role of early deposition of beta-amyloid (Aβ) in initiating multiple molecular and cellular events which culminate in death of brain neurons [1]. These studies identified common pathways by which misfolded protein aggregates induce injury, such as changes in brain calcium levels, mitochondrial dysfunction, oxidative stress, as well as microglial toxicity. The translational significance of this approach is the notion that removal of the aggressor may prevent consequential degeneration. However, to date, this therapeutic approach failed in clinical trials. A different, yet complementary therapeutic approach might be to strengthen the brain's intrinsic protective mechanisms in order to improve its defense against the toxic consequences of Aβ and of other misfolded protein aggregates. Surprisingly, there is severe lack of knowledge concerning the means by which the brain protects itself from degeneration.

Is there any evidence supporting the view that the brain possesses means of neuroprotection? One indication for their existence is the exceptionally large temporal gap between amyloid accumulation in the brain, and frank neurodegeneration [2]. Moreover, the large variance between patients who exhibit a heavy Aβ burden but develop early- versus late- AD suggests indeed the existence of significant individual differences in their brain protective mechanisms.

A major cellular candidate in providing neuroprotection is the pool of resident brain neural precursor/stem cells (NPC). NPCs are known for their powerful therapeutic immune-regulatory and neurotrophic properties [3,4]. NPC transplantation into the CNS has shown beneficial effects in multiple pre-clinical models of disease. Transplanted NPCs induce multiple bystander therapeutic effects to (1) ameliorate injurious neuro-inflammation, (2) protect neighboring brain cells from injury and degeneration, and (3) facilitate endogenous repair processes. Transplanted NPCs attenuate immune mediated tissue injury by modulating dendritic cell function, regulating microglial activity, interfering with multiple cytokine signaling and inhibiting T cell activation. Transplanted NPCs protect brain neurons from apoptosis, promote axon growth and remyelination. Studies in animal models of AD showed that transplanted NPCs improve memory impairment [5], in association with induction of BDNF-enhanced synaptogenesis, attenuation of neuro-inflammation (including inhibition of microgliosis, reduction of pro-inflammatory cytokine expression and of the TLR4 pathway), improvement in Aβ deposition/ clearance ratio, improvement in mitochondrial function, and enhancement of endogenous neurogenesis.

The apparent therapeutic properties of transplanted NPCs underscore the question whether resident brain NPCs have a role in regulating the progression of disease pathology in AD, and specifically in protecting the brain from degeneration.

Using the 5xFAD transgenic mouse model of AD we studied the therapeutic functions of resident brain NPCs at unique time points in the development of AD pathology [6]. These mice express a cassette of 5 mutated human genes causing familial AD. First, we confirmed that at age 2 months, these mice exhibit no overt pathology, at age 1 year they display significant loss of cortical neurons, and that age 7 months is a critical time point of heavy amyloid deposition and gliosis but prior to neurodegeneration. Analysis of resident NPC functional properties in 7-months old mice showed substantial dysfunction as indicated by both *ex vivo* and *in vivo* assays. Freshly isolated NPCs exhibited reduced expansion rate, and diminished immune-modulatory and trophic properties *ex-vivo*. Moreover, in spite of normal basal rate of NPC turnover / neurogenesis, there was slowed recovery of the NPC population in the subventricular zone following intra-cerebroventricular infusion of Cytosine-arabinoside, Also, there was a markedly reduced migratory response of subventricular zone NPCs to a Lysolecithin-induced lesion in the Corpus-Callosum *in vivo*. Importantly, these *ex-vivo* and *in-vivo* functions were fully preserved in two-months old AD mice, a time-point prior to Alzheimer's-specific pathological changes. The dysfunctional properties were reversible in NPCs derived from seven-months old AD mice following *in-vitro* expansion and were reproduced in wild type NPC by addition of Aβ peptide. Dysfunctional properties were associated with reduced expression of key genes involved in NPC proliferative and migratory responses, including fibroblast-growth-factor receptor-1, c-MET and HES1.

This work shows for the first time dysfunction of resident brain NPC in AD mice, which is acquired in an age-dependent manner, and is induced by the pathological Alzheimer's brain environment at a critical time point prior to neurodegeneration.

**Figure 1:**
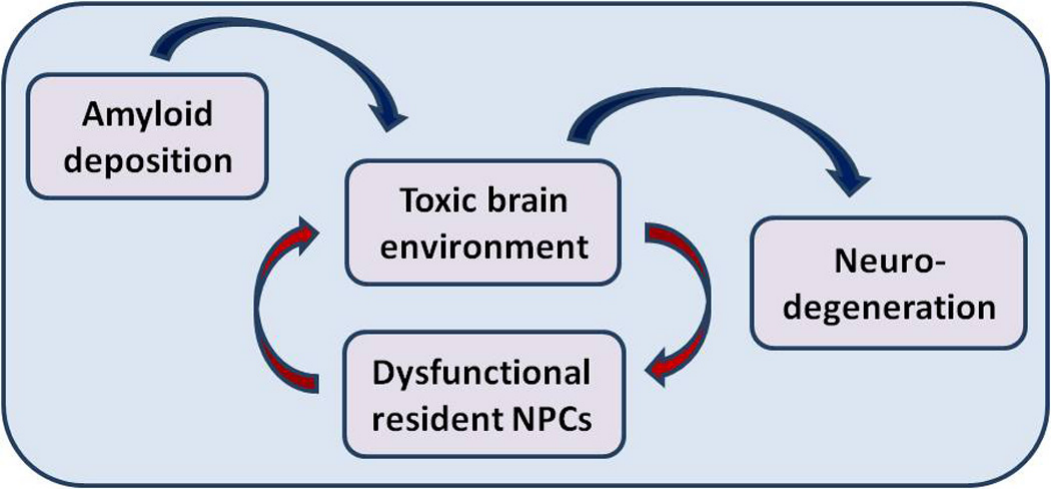
Environmental-induced functional failure of resident brain precursor cells may promote neurodegeneration in Alzheimer's disease.

In support of the long-existing concept that neurodegeneration in AD is related to lack of necessary trophic support for survival of brain synapses and neurons, these findings raise the possibility that failure of resident NPC to provide tissue support may be involved in promoting neurodegeneration. We suggest a model (Figure 1), where the AD brain environment induces resident NPC dysfunction, which in turn enhances the toxic effects of the pathologic environment. This may create a vicious cycle resulting in acceleration of neurodegeneration. To strengthen this hypothesis, it is necessary to examine further the functional properties of the various types of NPCs residing in multiple neurogenic niches (in addition to the subventricular zone studied here) and those distributed throughout the brain tissue, comprising ~5% of total cell number in the brain. It is necessary to study the bilateral interactions between resident NPCs and other brain cells involved in AD pathology, such as microglia [7] and reveal its molecular basis. This understanding, and the apparent reversibility of NPC dysfunction shown here, will create new valuable targets for therapeutic intervention in Alzheimer's disease.
